# Immune Response to a Synegeneic Rat Tumour: Evolution of Serum Cytotoxicity and Blockade

**DOI:** 10.1038/bjc.1973.151

**Published:** 1973-10

**Authors:** G. R. Flannery, P. J. Chalmers, J. M. Rolland, R. C. Nairn

## Abstract

The development of serum factors by Wistar rats during the growth of a syngeneic squamous cell carcinoma has been investigated to clarify the nature of the local lymphocyte anergy reported previously in this system. Sera from tumour bearing animals were tested for cytotoxicity against tumour cells by *in vitro* microassay, and their ability to inhibit cell mediated cytotoxicity was also studied. Serum cytotoxicity was first detected after 2 weeks of tumour growth, reaching a peak at 4 weeks and then declining. Inhibitory activity was found only in the sera of animals with advanced tumours. Anti-tumour antibody either in the sera or bound to tumour cells was not detected by immunofluorescence techniques. No evidence of general immunological debilitation was found, the animals showing normal immune responses to sheep erythrocytes and killed *Brucella abortus* organisms throughout tumour growth. Serum inhibitory factors may be responsible for the decline in anti-tumour immunoreactivity and the local lymphocyte anergy observed in tumour bearing hosts.


*Br. J. Cancer* (1973) **28**, 293

### IMMUNE RESPONSE TO A SYNGENEIC RAT TUMOUR: EVOLUTION OF SERUM CYTOTOXICITY AND BLOCKADE

G. R. FLANNERY, P. J. CHALMERS, J. M. ROLLAND AND R. C. NAIRN

*From the Department of Pathology, Monash University Medical School, Melbourne, Victoria, Australia*, 3181

Received 3 May 1973. Accepted 20 June 1973

**Summary.—The development of serum factors by Wistar rats during the growth of a syngeneic squamous cell carcinoma has been investigated to clarify the nature of the local lymphocyte anergy reported previously in this system. Sera from tumour bearing animals were tested for cytotoxicity against tumour cells by *in vitro* micro-assay, and their ability to inhibit cell mediated cytotoxicity was also studied. Serum cytotoxicity was first detected after 2 weeks of tumour growth, reaching a peak at 4 weeks and then declining. Inhibitory activity was found only in the sera of animals with advanced tumours. Anti-tumour antibody either in the sera or bound to tumour cells was not detected by immunofluorescence techniques. No evidence of general immunological debilitation was found, the animals showing normal immune responses to sheep erythrocytes and killed *Brucella abortus* organisms throughout tumour growth. Serum inhibitory factors may be responsible for the decline in anti-tumour immunoreactivity and the local lymphocyte anergy observed in tumour bearing hosts.**

LOCAL lymphocyte anergy has been reported previously in a study of the development of cell mediated immunity to a syngeneic squamous cell carcinoma in inbred Wistar rats (Flannery *et al.*, 1973). Regional node lymphocytes, cytotoxic to tumour cells early in tumour growth, lose this ability as the tumour progresses and become totally unresponsive whereas spleen and blood lymphocytes retain a significant degree of cytotoxicity. It has been shown that factors present in the sera of tumour bearing hosts interfere with lymphocyte-mediated tumour-cell destruction (reviewed by Hellström and Hellström, 1971). These factors include excess tumour antigen (Alexander, 1970), anti-tumour antibody (Hellström and Hellström, 1970) and antigen–antibody complexes (Baldwin, Price and Robins, 1972; Sjögren *et al.*, 1972). We have studied the evolution of serum cytotoxicity and blockade throughout the period of tumour growth in rats to investigate any relationship between these factors and regional anergy.

### MATERIAL AND METHODS

*Animals and tumour*.—The growth of a transplantable squamous cell carcinoma in Wistar rats and the use of tumour cell suspensions for inoculation and cytotoxicity assays have been described previously (Flannery *et al.*, 1973).

*Tumour growth* in vivo.—The pattern of tumour growth *in vivo* was studied in 2 groups of animals: (i) Rats inoculated subcutaneously in the medial aspect of the right thigh with $10^3$ viable tumour cells, previously shown to produce tumours in all animals and death in 8–10 weeks. Groups of 4 animals were killed 2, 4, 6 and 8 weeks after inoculation. (ii) Rats inoculated similarly with $10^4$ viable tumour cells, previously shown to kill all animals in 6–8 weeks. Groups of 5 animals were killed weekly after tumour inoculation and the volumes of the primary tumours were determined (Flannery *et al.*, 1973).

### *Immunological studies* in vitro

*Serum cytotoxicity*.—Sera were tested for *in vitro* complement-dependent cytotoxicity to tumour cells by a modification of the method of Bloom (1970). Tumour cells were plated on to microtitration plates (No. 3034, Falcon Plastics Co.) as described for the lymphocytotoxicity test (Flannery *et al.*, 1973). Medium was removed from each well after 24 hours at 37°C and 5 $\mu$l of test, or control normal serum, heat-inactivated by heating at 56°C for 30 min, was added by micropipette. After 30 min at 37°C, 5 $\mu$l of a 1 in 2 dilution of reconstituted, freeze dried guinea-pig complement (Wellcome Laboratories) was added and the plates reincubated at 37°C for 5 hours. Plates were then fixed and stained with Leishman's stain and the number of cells adhering to the bottom of each well was counted. Cytotoxicity was expressed as the percentage reduction in the mean number of surviving tumour cells in test ($Nt$) versus control ($Nc$) wells, *i.e.* cytotoxicity =

$$\frac{Nc - Nt}{Nc} \times 100.$$

*Serum blocking activity.*—The ability of sera from tumour bearing animals to inhibit lymphocyte attack on tumour cells was assessed by a modification of the method of Hellström *et al.* (1971). For the lymphocytotoxicity test (Flannery *et al.*, 1973), wells containing approximately 50 tumour cells were used in all tests and at least 5 replicate wells were counted. Spleen lymphocytes were obtained from an immunized syngeneic donor and stored with 10% dimethylsulphoxide in liquid nitrogen before use. Although such storage is sometimes associated with impairment of immunoreactivity of lymphocytes, in this particular instance, in comparison with similarly stored normal lymphocytes, they exhibited a cytotoxicity of approximately 25%. Before the addition of the lymphocytes, 10 $\mu$l of the test serum (diluted 1 in 5 with medium 199) was added to each well containing tumour cells, incubated at 37°C for 45 min and removed by washing. Blocking ability was expressed as the percentage reduction in cytotoxicity in test ($Ct$) versus control ($Cc$) wells *i.e.* blocking =

$$\frac{Cc - Ct}{Cc} \times 100.$$

Student's $t$-test was used to assess the statistical significance of differences between cytotoxicity means, and a difference was considered significant at the $P < 0{\cdot}05$ level.

*Membrane immunofluorescence*—Membrane staining of viable tumour cells in suspension treated with the rat sera was carried out with appropriate controls by sandwich immunofluorescence described elsewhere (Nairn, 1969) using an apparatus designed to facilitate simultaneous handling of up to 40 sera (Nairn, Cusdin and McNaughtan, 1971). A fluorescein labelled rabbit anti-rat globulin with a fluorescein to protein molar ratio of 4·3 : 1 was used at a globulin concentration of 0·7 g/100 ml. This conjugate had been absorbed with human liver powder, bovine liver homogenate and well washed rat erythrocytes and had activity against IgG and IgM. By itself it gave no fluourescent staining of rat tumour cells or thymocytes.

*Frozen sections.*—Fresh blocks of tumour, up to 5 mm square × 2 mm thick, were excised from each rat killed 2, 4, 6 and 8 weeks after inoculation with $10^3$ tumour cells. Blocks were snap frozen in a liquid nitrogen–isopentane mixture and stored at − 70°C. Sections, 6 $\mu$m, were cut and stained by direct immunofluorescence using standard controls (Nairn, 1969), and the fluorescein labelled rabbit anti-rat globulin described above.

*Impression films.*—Frozen tumour blocks were thawed to room temperature and impression films made by pressing the tissue against gelatinized glass slides (Nairn, 1969). Films were stained by direct immunofluorescence as for frozen sections.

*Antibody responses to other antigens.*—Four parallel groups, each of 4 animals were inoculated with $10^3$ tumour cells and injected intraperitoneally 1, 3, 5 or 7 weeks later with 0·1 ml of a 50% suspension of sheep red cells (Commonwealth Serum Laboratories) and 0·15 ml of a suspension of killed *Brucella abortus* (Ring test antigen, Commonwealth Serum Laboratories). Animals were bled and killed 7 days after antigen injection. Antibody activity to sheep red cells was determined by haemagglutination assays with 2·5% red cells in microtitre trays. Response to *Brucella* was also determined by agglutination assay in microtitre trays with a 1 in 250 dilution of the haemotoxylin stained *Brucella* preparation.

### RESULTS

### *Tumour growth*

The pattern of tumour growth in both groups of animals was similar.

Tumours became palpable 2–4 weeks after inoculation and then increased rapidly in volume, reaching a mean size of 17·1 ± 8·0 $cm^3$ in the rats inoculated with $10^3$ cells, and 11·6 ± 4·6 $cm^3$ after $10^4$ cells. Metastases were observed microscopically in the lungs of some animals when tumours became palpable, and of all animals by Week 6.

*Serum cytotoxicity*

The time course of serum cytotoxicity is shown in Fig. 1, in which mean values are plotted. Reactivity reached a peak

FIG. 1.—Evolution of serum cytotoxicity during tumour growth in rats after inoculation with $10^3$ tumour cells (●——●, means of 4 animals) and $10^4$ tumour cells (●- - -●, means of 5 animals). Means ± standard error.

in both groups of animals at Week 4, after which it declined and was not significant beyond Week 6. No cytotoxicity was demonstrable in the absence of complement.

*Serum blockade of lymphocyte cytotoxicity*

The ability of sera from tumour bearing animals to inhibit the lymphocytotoxicity reaction is summarized in Tables I and II. The sera from animals inoculated with $10^3$ tumour cells showed no inhibition until Week 8, by which time all animals tested were positive; an exception was the serum of one animal which was

TABLE I.—*Development of Humoral Blocking Reactivity by Tumour Bearing Rats* ($10^3$ *Tumour Cell Inoculum*)

| Week | 2 | 4 | 6 | 8 |
|---|---|---|---|---|
| Reduction of | 0 | 0 | 0 | 52* |
| lymphocytotoxicity | 7 | 4 | 0 | 60† |
| (%) | 9 | 12 | 7 | 72† |
| | 15 | 42* | 49 | ... |

... Not tested.

* Significant, $P < 0·05$.

† Significant, $P < 0·01$.

TABLE II.—*Development of Humoral Blocking Reactivity by Tumour Bearing Rats* ($10^4$ *Tumour Cell Inoculum*)

| Week | 1 | 2 | 3 | 4 | 5 | 6 | 7 |
|---|---|---|---|---|---|---|---|
| Reduction of | 0 | 0 | 0 | 0 | 0 | 0 | 0 |
| lymphocytotoxicity | 0 | 0 | 0 | 0 | 0 | 0 | 19 |
| (%) | 0 | 1 | 18 | 2 | 0 | 0 | 93† |
| | 10 | 2 | 33 | 7 | 0 | 0 | 100* |
| | 12 | 57 | 36 | 23 | 5 | 29 | 100† |

* Significant, $P < 0·05$.

† Significant, $P < 0·01$.

inhibitory at Week 4. Animals inoculated with $10^4$ tumour cells showed no serum inhibitory activity until Week 7, when 3 of 5 sera tested were positive.

*Antibodies to tumour*

Despite demonstrable *in vitro* cytotoxicity of the sera from many tumour bearing animals against tumour cells, no anti-tumour cell membrane antibodies were detected by membrane immunofluorescence, which is doubtless a less sensitive index of antibody binding. No antibody pre-bound to cells was demonstrable in either frozen sections or impression films of tumour.

*Response to sheep erythrocyte and* Brucella abortus *antigens*

The antibody responses of tumour bearing rats to sheep red blood cells and *Brucella abortus* during tumour growth are summarized in Table III. The results show that the levels of response did not differ significantly at any time from those of control, non-tumour bearing rats. An apparent deviation in the response to

TABLE III.—*Reactivity of Tumour Bearing Rats against Sheep Red Blood Cells and Killed* Brucella abortus *Organisms. Means of* 4 *animals*

| | Log$_2$ titre (range and mean) | |
|---|---|---|
| Week | Sheep red cells | *Brucella abortus* |
| 2 | 8–10 (9) | 11–13 (12) |
| 4 | 8–9 (8) | 11–14 (13) |
| 6 | 6–8 (8) | 10–13 (12) |
| 8 | 1*–9 (6) | 11–14 (13) |
| Normal controls (10 animals) | 8–10 (9) | 11–14 (13) |

* Titre of 1 rat only below normal range.

sheep red cells at Week 8, due to a low titre in one rat, was not statistically significant.

### DISCUSSION

We have studied the development, in tumour bearing hosts, of serum factors capable of tumour cell destruction and factors able to inhibit lymphocyte cytotoxicity. Serum mediated cytotoxicity became apparent early in tumour growth and then declined. The size of the initial tumour inoculum did not alter this pattern. A similar time course has been reported for the development of lymphocytotoxicity in these animals (Flannery *et al.*, 1973).

The complement dependence of the serum cytotoxicity and the work of others (Takasugi and Hildemann, 1969; Ankerst, 1971) implicate antibodies in this reaction. The serum concentration of such antibodies was in any case low, as no activity against tumour cell membrane antigens was detected by immunofluorescence. In view of the similar decline in lymphocyte cytotoxicity, the observed reduction in serum reactivity may have been due to a lymphocyte impairment, either as a direct B cell deficiency or as a defect in T cell collaboration. A general immunological debilitation, however, was not demonstrated: normal antibody responses were maintained in tumour bearing animals to *Brucella abortus*, a measure primarily of B cell function (Crewther and Warner, 1972; Hard, G. C. personal communication) and to sheep erythrocytes, a function requiring T and B cell co-operation (Claman and Chaperon, 1969). An alternative hypothetical explanation, which has not yet been studied, would be a shift in the production of IgM antibody to less lytic IgG antibody during maturation of the immune response (Takasugi and Hildemann, 1969; Ankerst, 1971).

Inhibition of lymphocyte cytotoxicity by sera of tumour bearing animals was seen in later stages of tumour growth. We have not yet investigated the nature of the serum blocking factors in our system, but their occurrence may be another explanation for the decline in antitumour immunoreactivity. The late detection of blocking may have been due to the relatively low sensitivity of the technique used, as others (Sjögren and Borum, 1971) have reported the appearance of blocking factors throughout tumour growth. Alternatively, the appearance of blocking factors and the disappearance of cytotoxic antibody may be associated with the concurrent growth of metastases. The possibility of " unblocking factors " (Hellström and Hellström, 1970; Bansal and Sjögren, 1971) earlier in tumour growth has not been investigated in our system.

*In vivo*, blocking factors might act on effector lymphocytes or on tumour cells. Circulating lymphocytes will be more accessible to humoral factors and specific depression of lymphocytes has been described (Field and Caspary, 1972). Currie and Basham (1972) have demonstrated that washing of lymphocytes from cancer patients increased their cytotoxic activity; subsequent incubation of the prewashed lymphocytes with autologous serum abolished this reactivity. The anergy of local lymphocytes in our system (Flannery *et al.*, 1973) may well be due to lymphocyte inhibition of this type. Blocking factors have been detected by us and by others (*e.g.* Hellström *et al.*, 1971) which bound to target cells, and eluates from tumour cell suspensions have been shown to possess *in vitro* and *in vivo*

blocking reactivity (Bansal, Hargreaves and Sjögren, 1972; Ran and Witz, 1972; Sjögren *et al.*, 1972). Others (Hellström and Hellström, 1969; Cohen, Millar and Ketcham, 1972) have demonstrated blocking by incubation of tumour cells with serum but failed to show inhibition by similar incubation of lymphocytes. Such tumour bound factors cannot account for the lymphocyte anergy we observed and their *in vivo* significance is unclear. Evidence that inhibitory factors may be antigen–antibody complexes (Sjögren *et al.*, 1971, 1972; Baldwin *et al.*, 1972) suggests that they might be able to act on lymphocytes whilst bound to tumour cells (Sjögren *et al.*, 1972). In our studies, binding of antibodies to lymphocytes or tumour cells was not detected by immunofluorescence, presumably due, if present, to their low *in vivo* levels.

We have shown a local lymphocyte anergy and the presence in serum of factors capable of inhibiting lymphocytotoxicity. If blocking factors are responsible for *in vivo* loss of lymphocyte reactivity, there must be some local component to account for the early development of anergy in the regional nodes. This local factor seems most likely to be excess tumour antigen which may then pass into the serum, perhaps complexed with antibody, as the inhibitor we have detected. The isolation and characterization of this factor(s) are proceeding.

G. R. Flannery and P. J. Chalmers are Monash University Graduate Scholars and the work is part of their Ph.D. projects. It is supported by grants from the Anti-Cancer Council of Victoria and the National Health and Medical Research Council. We thank Miss H. Goldsmith for technical assistance.

### REFERENCES

ALEXANDER, P. (1970) Prospects for Immunotherapy of Cancer: Experience in Experimental Systems. *Br. med. J.*, iv, 484.

ANKERST, J. (1971) Demonstration and Identification of Cytotoxic Antibodies and Antibodies Blocking the Cell-mediated Antitumor Immunity against Adenovirus Type 12-induced Tumors. *Cancer Res.*, **31**, 997.

BALDWIN, R. W., PRICE, M. R. & ROBINS, R. A. (1972) Blocking of Lymphocyte-mediated Cytotoxicity for Rat Hepatoma Cells by Tumour-specific Antigen–antibody Complexes. *Nature, New Biol.*, **238**, 185.

BANSAL, S. C. & SJÖGREN, H. O. (1971) " Unblocking " Serum Activity *in vitro* in the Polyoma System may Correlate with Antitumour Effects of Antiserum *in vivo*. *Nature, New Biol.*, **233**, 76.

BANSAL, S. C., HARGREAVES, R. & SJÖGREN, H. O. (1972) Facilitation of Polyoma Tumor Growth in Rats by Blocking Sera and Tumor Eluate. *Int. J. Cancer*, **9**, 97.

BLOOM, E. T. (1970) Quantitative Detection of Cytotoxic Antibodies Against Tumor-specific Antigens of Murine Sarcoma Induced by 3-methylcholanthrene. *J. natn. Cancer Inst.*, **45**, 443.

CLAMAN, H. N. & CHAPERON, E. A. (1969) Immunological Complementation between Thymus and Marrow Cells—A Model for the Two-cell Theory of Immunocompetence. *Transplantn Rev.*, **1**, 92.

COHEN, A. M., MILLAR, R. C. & KETCHAM, A. S. (1972) Host Immunity to a Growing Transplanted Methylcholanthrene-induced Guinea Pig Sarcoma. *Cancer Res.*, **32**, 2421.

CREWTHER, P. & WARNER, N. L. (1972) Serum Immunoglobulins and Antibodies in Congenitally Athymic (Nude) Mice. *Aust. J. exp. Biol. med. Sci.*, **50**, 625.

CURRIE, G. A. & BASHAM, C. (1972) Serum-mediated Inhibition of the Immunological Reactions of the Patient to his own Tumour: a Possible Role for Circulating Antigen. *Br. J. Cancer*, **26**, 427.

FIELD, E. J. & CASPARY, E. A. (1972) Lymphocyte Sensitization in Advanced Malignant Disease: a Study of Serum Lymphocyte Depressive Factor. *Br. J. Cancer*, **26**, 164.

FLANNERY, G. R., CHALMERS, P. J., ROLLAND, J. M. & NAIRN, R. C. (1973) Immune Response to a Sygeneic Rat Tumour: Development of Regional Node Lymphocyte Anergy. *Br. J. Cancer*, **28**, 118.

HELLSTRÖM, I. & HELLSTRÖM, K. E. (1969) Studies on Cellular Immunity and its Serum-mediated Inhibition in Moloney-virus-induced Mouse Sarcomas. *Int. J. Cancer*, **4**, 587.

HELLSTRÖM, I. & HELLSTRÖM, K. E. (1971) Cellular Immunity and Blocking Antibodies to Tumors. *J. Reticuloendothel. Soc.*, **10**, 131.

HELLSTRÖM, I., SJÖGREN, H. O., WARNER, G. A. & HELLSTRÖM, K. E. (1971) Blocking of Cell-mediated Tumor Immunity by Sera from Patients with Growing Neoplasms. *Int. J. Cancer*, **7**, 226.

HELLSTRÖM, K. E. & HELLSTRÖM, I. (1970) Immunological Enhancement as Studied by Cell Culture Techniques *A. Rev. Microbiol.*, **24**, 373.

NAIRN, R. C. (1969) *Fluorescent Protein Tracing*, 3rd Ed. Edinburgh: Livingstone.

NAIRN, R. C., CUSDIN, C. H. & MCNAUGHTAN, J. (1971) Partial Mechanization of Membrane Immunofluorescent Staining. *Clin. & exp. Immunol.*, **8**, 835.

RAN, M. & WITZ, I. P. (1972) Tumor-associated Immunoglobulins. Enhancement of Syngeneic Tumors by IgG2-containing Tumor Eluates. *Int. J. Cancer*, **9**, 242.

SJÖGREN, H. O. & BORUM, K. (1971) Tumor-specific Immunity in the Course of Polyoma and Rous Tumor Development in Intact and Immuno-depressed Rats. *Cancer Res.*, **31**, 890.

SJÖGREN, H. O., HELLSTRÖM, I., BANSAL, S. C. & HELLSTRÖM, K. E. (1971) Suggestive Evidence that "Blocking Antibodies" of Tumor-bearing Individuals May be Antigen–antibody Complexes. *Proc. natn. Acad. Sci. U.S.A.*, **68**, 1372.

SJÖGREN, H. O., HELLSTRÖM, I., BANSAL, S. C., WARNER, G. A. & HELLSTRÖM, K. E. (1972) Elution of "Blocking Factors" from Human Tumors, Capable of Abrogating Tumor Cell Destruction by Specifically Immune Lymphocytes. *Int. J. Cancer*, **9**, 274.

TAKASUGI, M. & HILDEMANN, W. H. (1969) Regulation of Immunity toward Allogeneic Tumors in Mice. I. Effect of Antiserum Fractions on Tumor Growth. *J. natn. Cancer. Inst.*, **43**, 843.


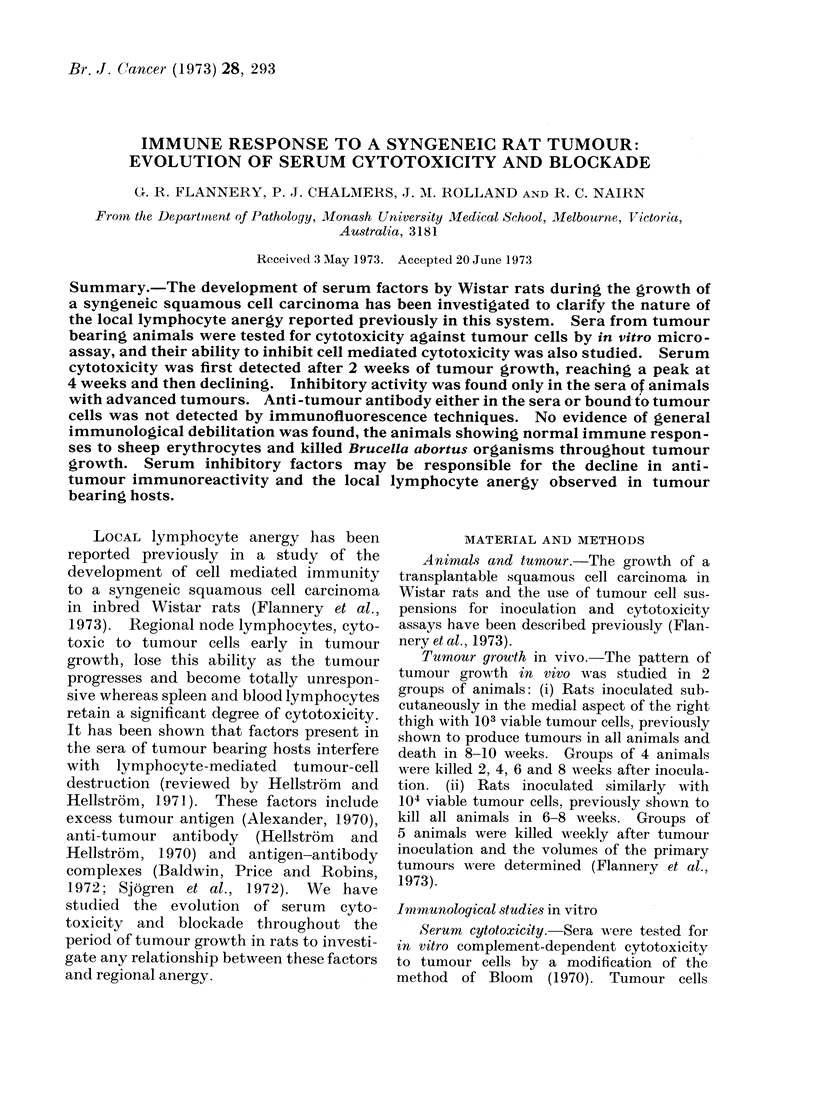

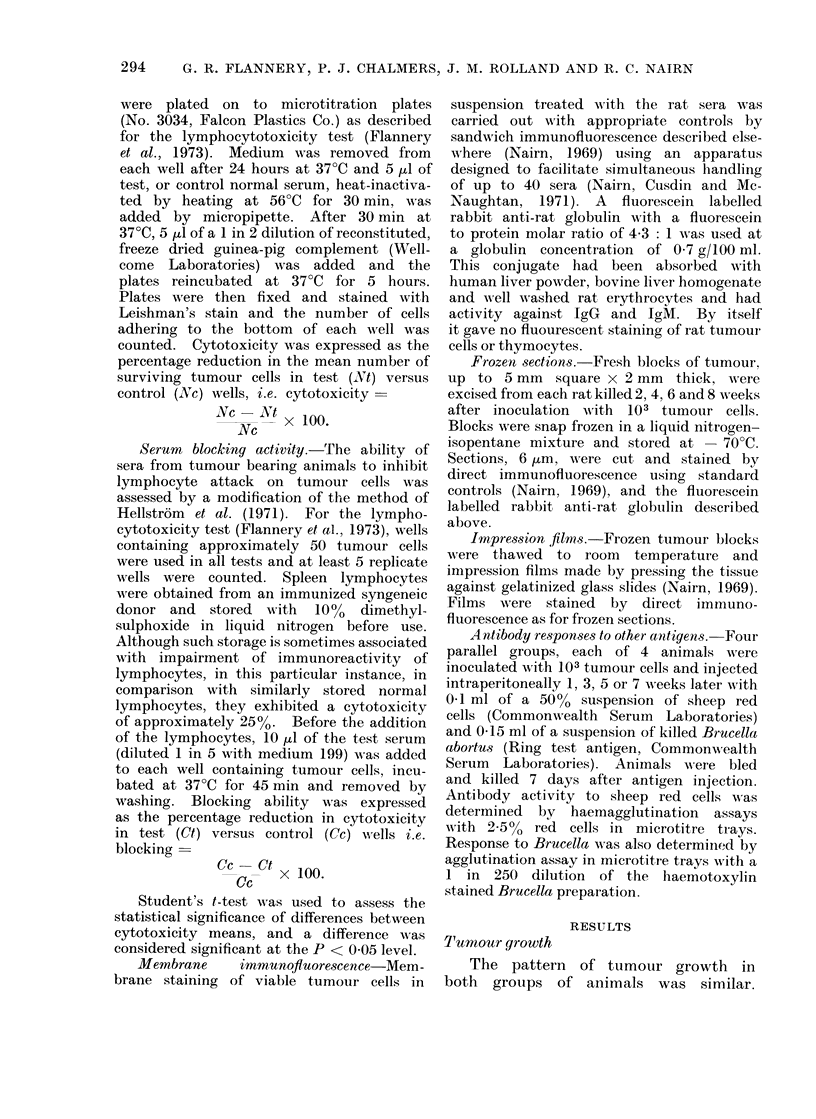

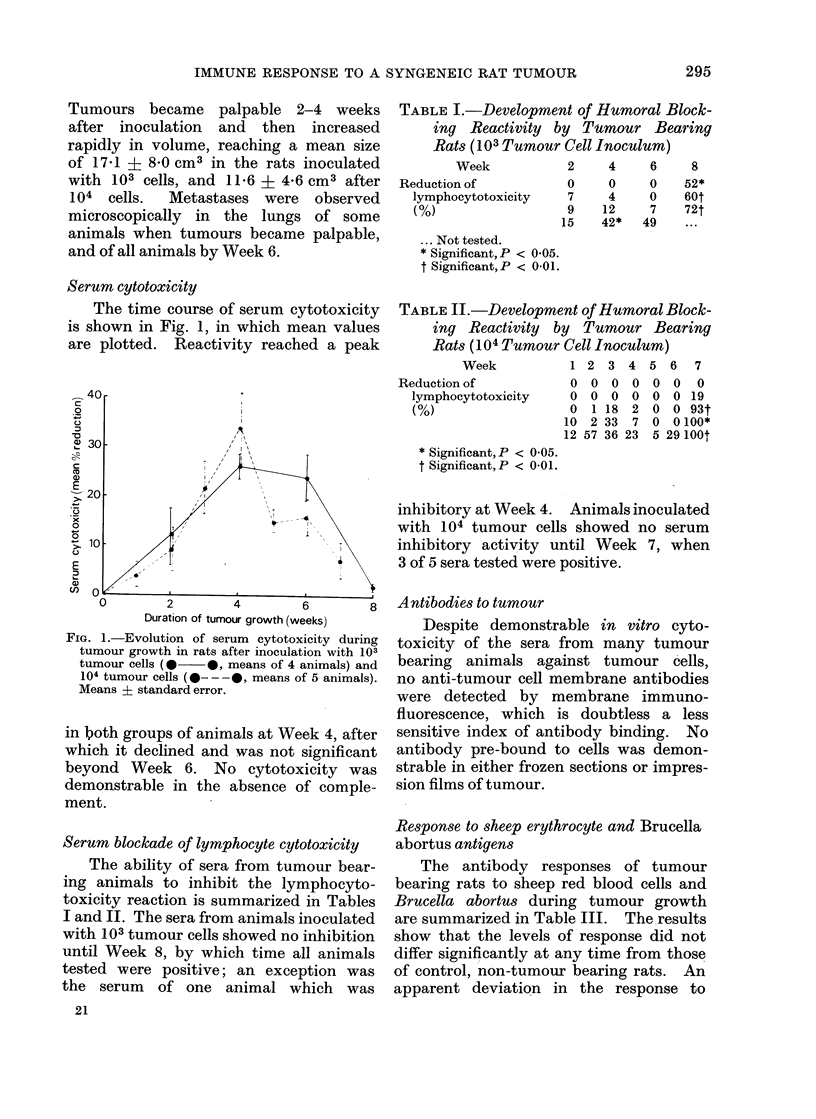

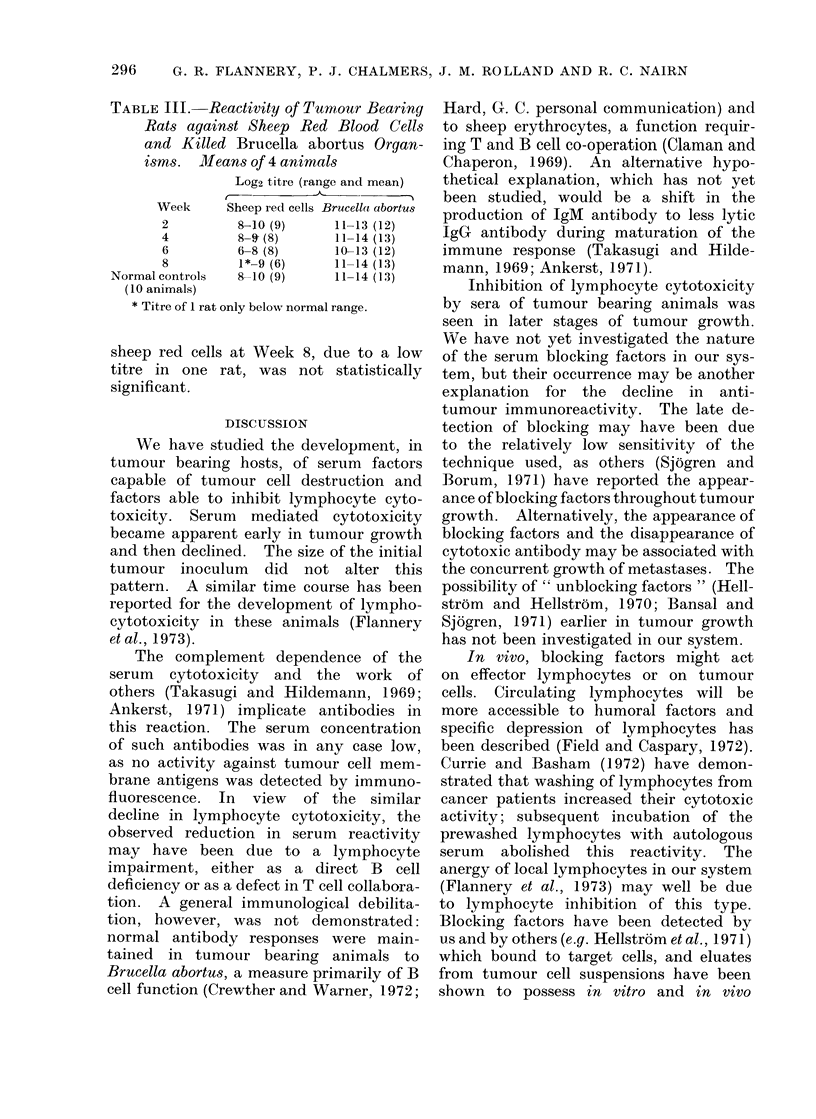

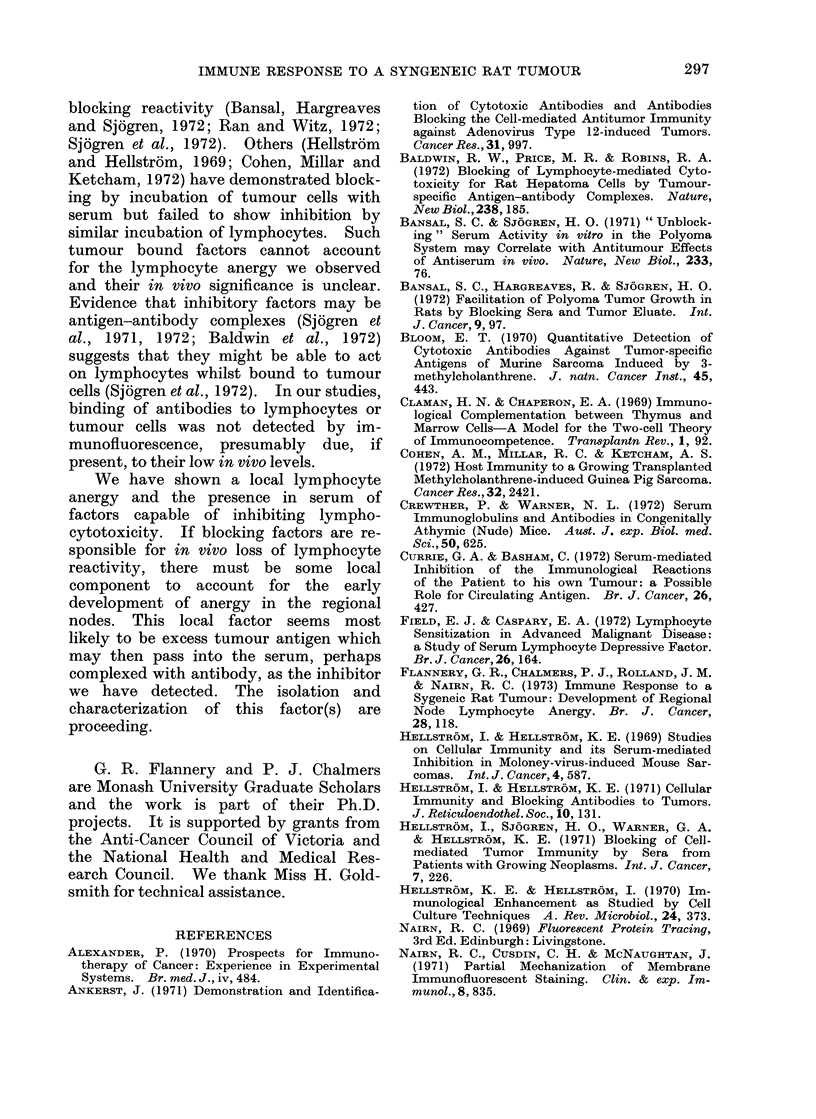

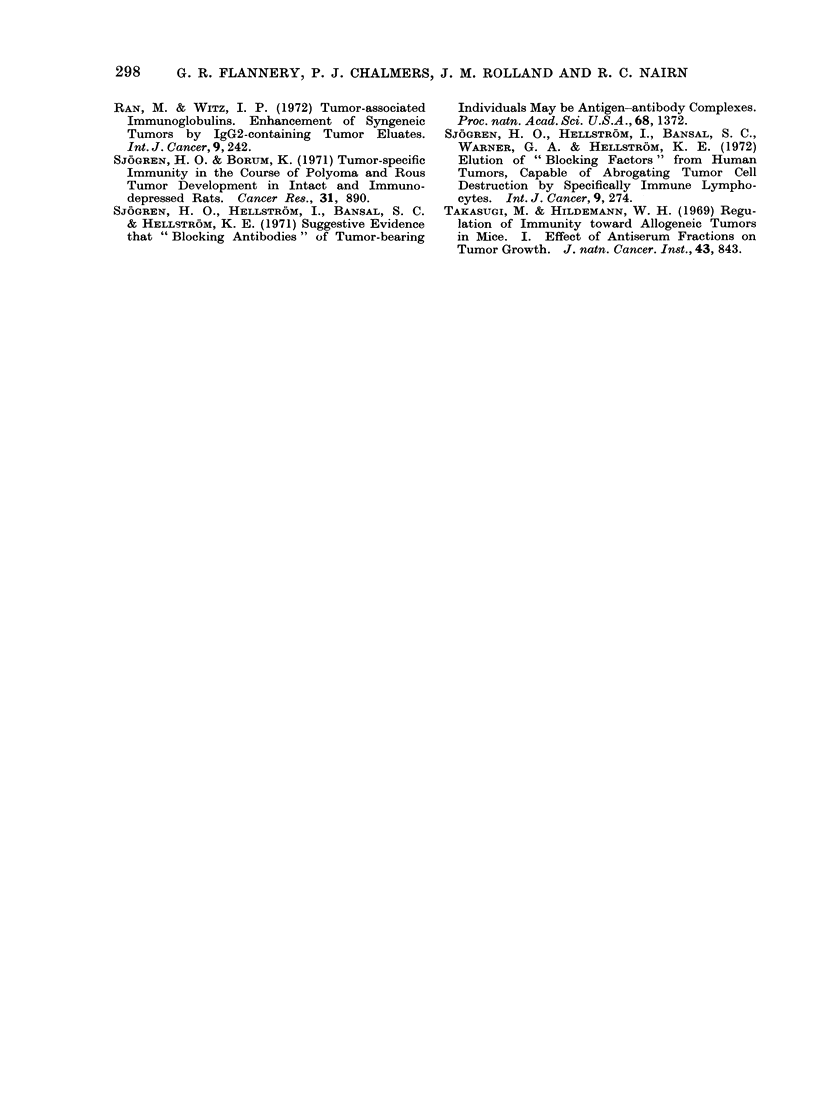


## References

[OCR_00525] Alexander P. (1970). Prospects for immunotherapy of cancer: experience in experimental systems.. Br Med J.

[OCR_00530] Ankerst J. (1971). Demonstration and identification of cytotoxic antibodies and antibodies blocking the cell-mediated antitumor immunity against adenovirus type 12-induced tumors.. Cancer Res.

[OCR_00537] Baldwin R. W., Price M. R., Robins R. A. (1972). Blocking of lymphocyte-mediated cytotoxicity for rat hepatoma cells by tumour-specific antigen-antibody complexes.. Nat New Biol.

[OCR_00557] Bloom E. T. (1970). Quantitative detection of cytotoxic antibodies against tumor-specific antigens of murine sarcomas induced by 3-methylcholanthrene.. J Natl Cancer Inst.

[OCR_00564] Claman H. N., Chaperon E. A. (1969). Immunologic complementation between thymus and marrow cells--a model for the two-cell theory of immunocompetence.. Transplant Rev.

[OCR_00569] Cohen A. M., Millar R. C., Ketcham A. S. (1972). Host immunity to a growing transplanted methylcholanthrene-induced guinea pig sarcoma.. Cancer Res.

[OCR_00575] Crewther P., Warner N. L. (1972). Serum immunoglobulins and antibodies in congenitally athymic (nude) mice.. Aust J Exp Biol Med Sci.

[OCR_00581] Currie G. A., Basham C. (1972). Serum mediated inhibition of the immunological reactions of the patient to his own tumour: a possible role for circulating antigen.. Br J Cancer.

[OCR_00588] Field E. J., Caspary E. A. (1972). Lymphocyte sensitization in advanced malignant disease: a study of serum lymphocyte depressive factor.. Br J Cancer.

[OCR_00594] Flannery G. R., Chalmers P. J., Rolland J. M., Nairn R. C. (1973). Immune response to a syngeneic rat tumour: development of regional node lymphocyte anergy.. Br J Cancer.

[OCR_00607] Hellström I., Hellström K. E. (1971). Cellular immunity and blocking antibodies to tumors.. J Reticuloendothel Soc.

[OCR_00603] Hellström I., Hellström K. E. (1969). Studies on cellular immunity and its serum mediated inhibition in Moloney-virus-induced mouse sarcomas.. Int J Cancer.

[OCR_00612] Hellström I., Sjögren H. O., Warner G., Hellström K. E. (1971). Blocking of cell-mediated tumor immunity by sera from patients with growing neoplasms.. Int J Cancer.

[OCR_00619] Hellström K. E., Hellström I. (1970). Immunological enhancement as studied by cell culture techniques.. Annu Rev Microbiol.

[OCR_00627] Nairn R. C., Cusdin C. H., McNaughtan J. (1971). Partial mechanization of membrane immunofluorescent staining techniques.. Clin Exp Immunol.

[OCR_00635] Ran M., Witz I. P. (1972). Tumor-associated immunoglobulins. Enhancement of syngeneic tumors by IgG2-containing tumor eluates.. Int J Cancer.

[OCR_00641] Sjögren H. O., Borum K. (1971). Tumor-specific immunity in the course of primary polyoma and Rous tumor development in intact and immunosuppressed rats.. Cancer Res.

[OCR_00649] Sjögren H. O., Hellström I., Bansal S. C., Hellström K. E. (1971). Suggestive evidence that the "blocking antibodies" of tumor-bearing individuals may be antigen--antibody complexes.. Proc Natl Acad Sci U S A.

[OCR_00657] Sjögren H. O., Hellström I., Bansal S. C., Warner G. A., Hellström K. E. (1972). Elution of "blocking factors" from human tumors, capable of abrogating tumor-cell destruction by specifically immune lymphocytes.. Int J Cancer.

[OCR_00663] Takasugi M., Hildemann W. H. (1969). Regulation of immunity toward allogeneic tumors in mice. I. Effect of antiserum fractions on tumor growth.. J Natl Cancer Inst.

